# Supplementary figures and images for: Functional study of two flexible regions of the hepatitis E virus ORF1 replicase
Source: PLoS One. 2026 Mar 10;21(3):e0343555. doi: 10.1371/journal.pone.0343555 (PMC12974834; doi:10.1371/journal.pone.0343555)

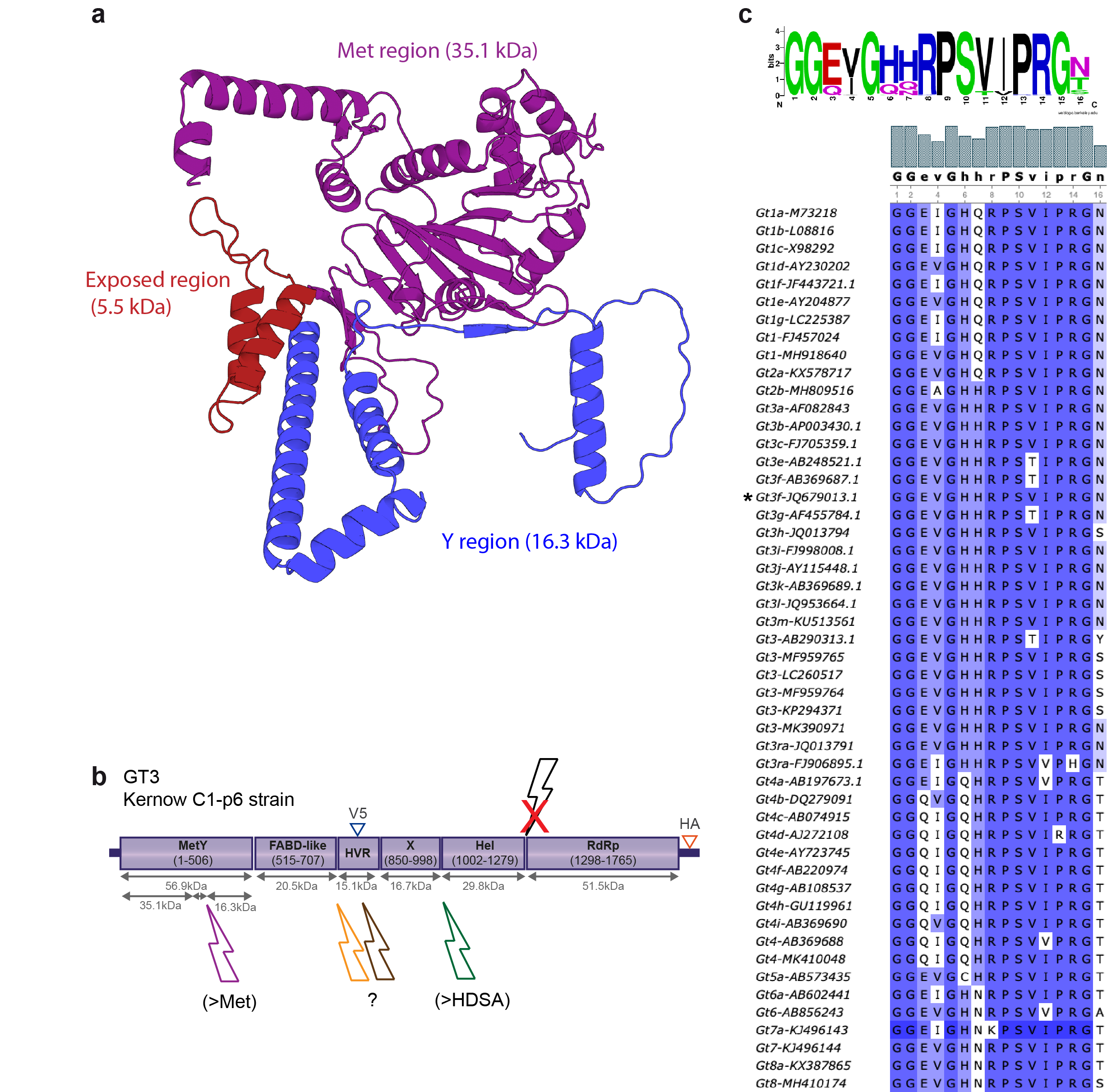

Supplement: S1 Fig — (TIF) [file pone.0343555.s001.tif]

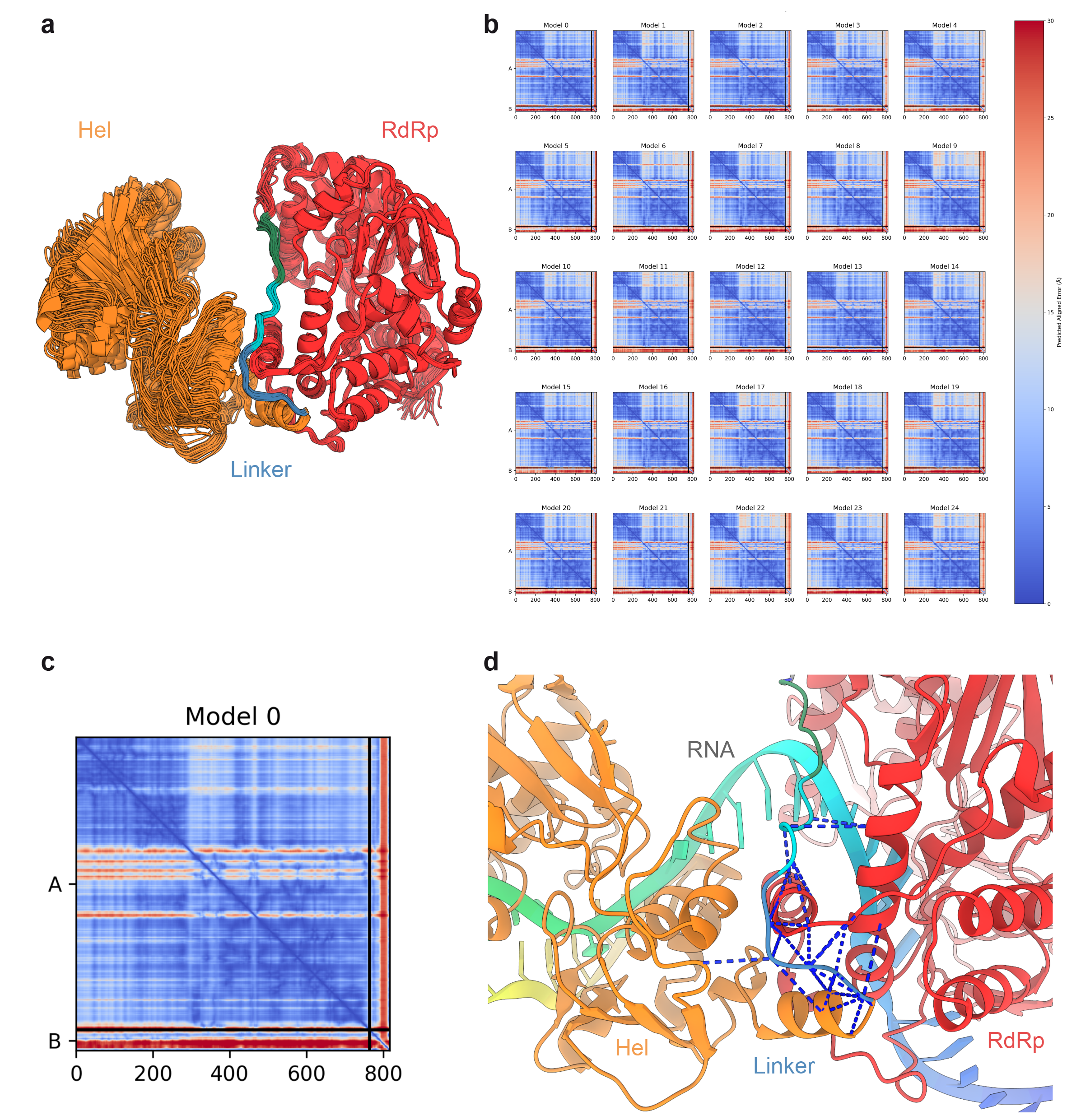

Supplement: S2 Fig — (TIF) [file pone.0343555.s002.tif]

Raw images Figure 2

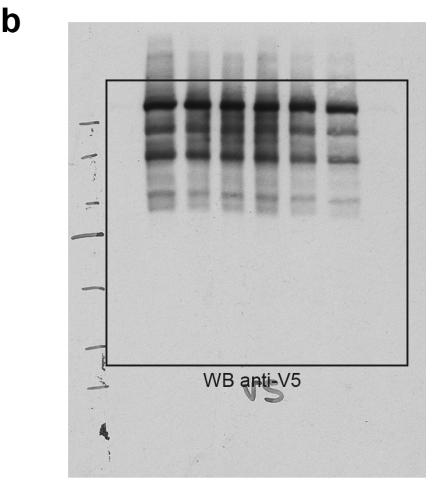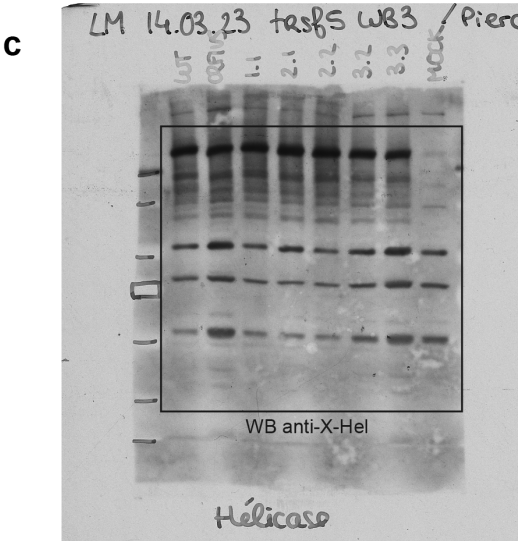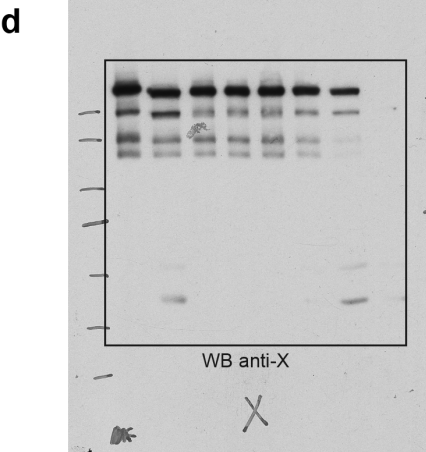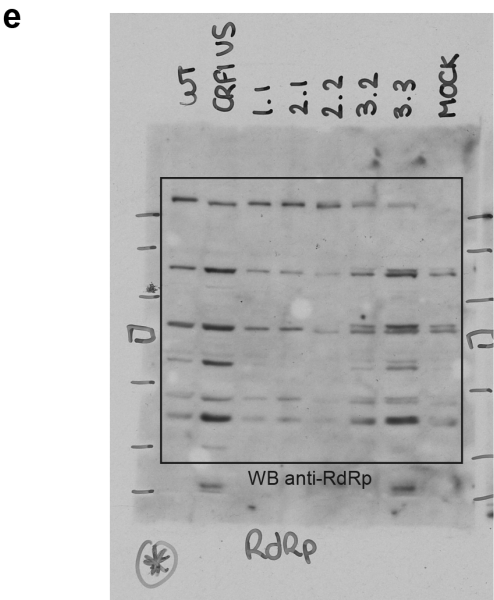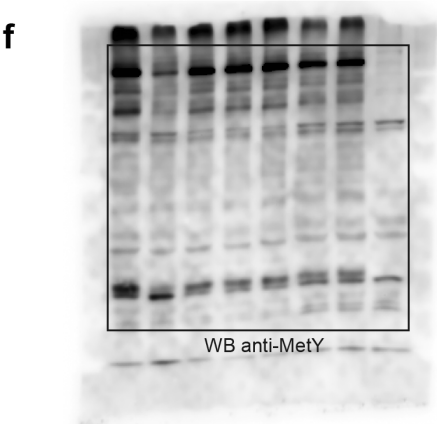

Raw images Figure 3

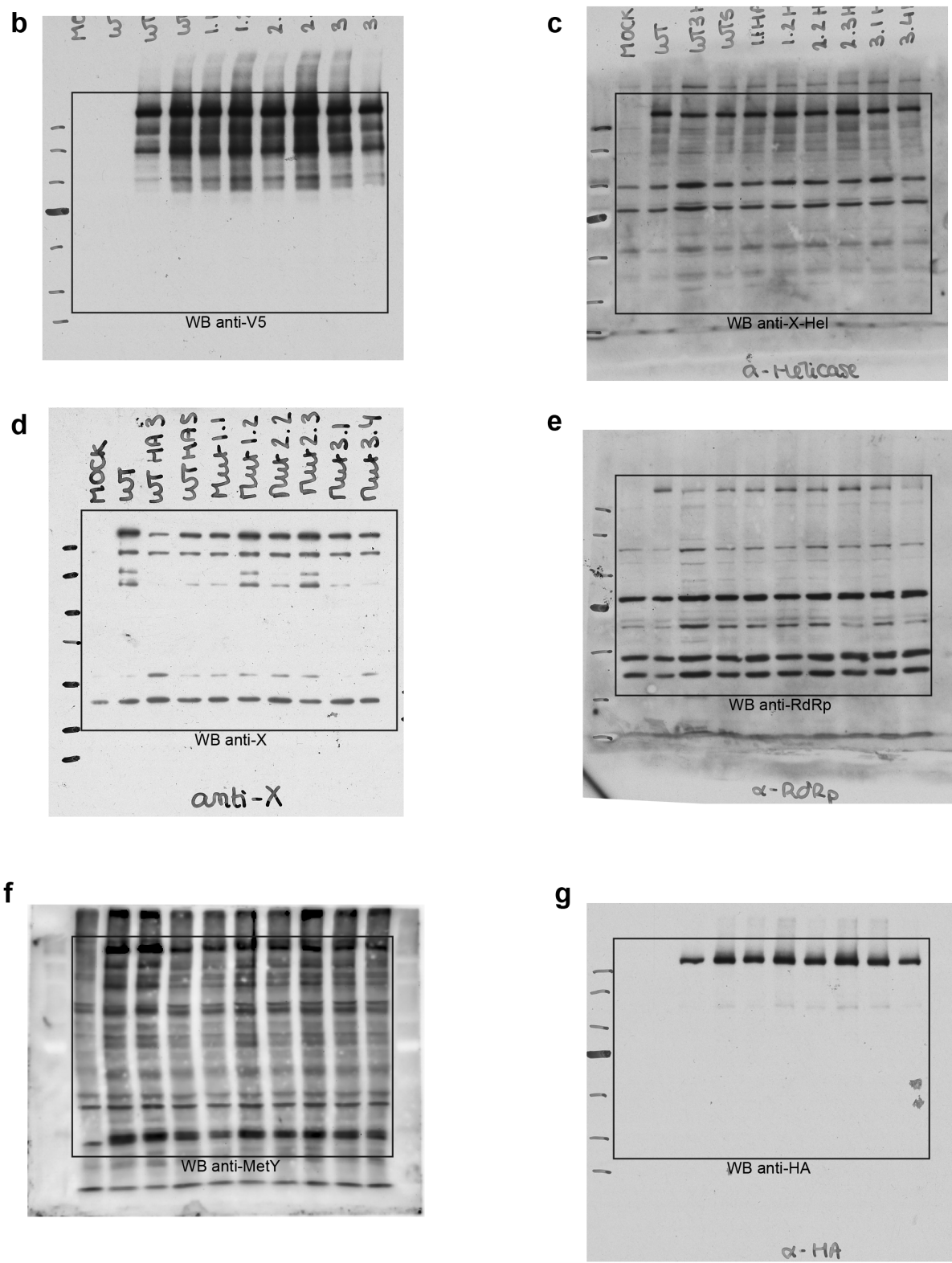

Raw images Figure 4

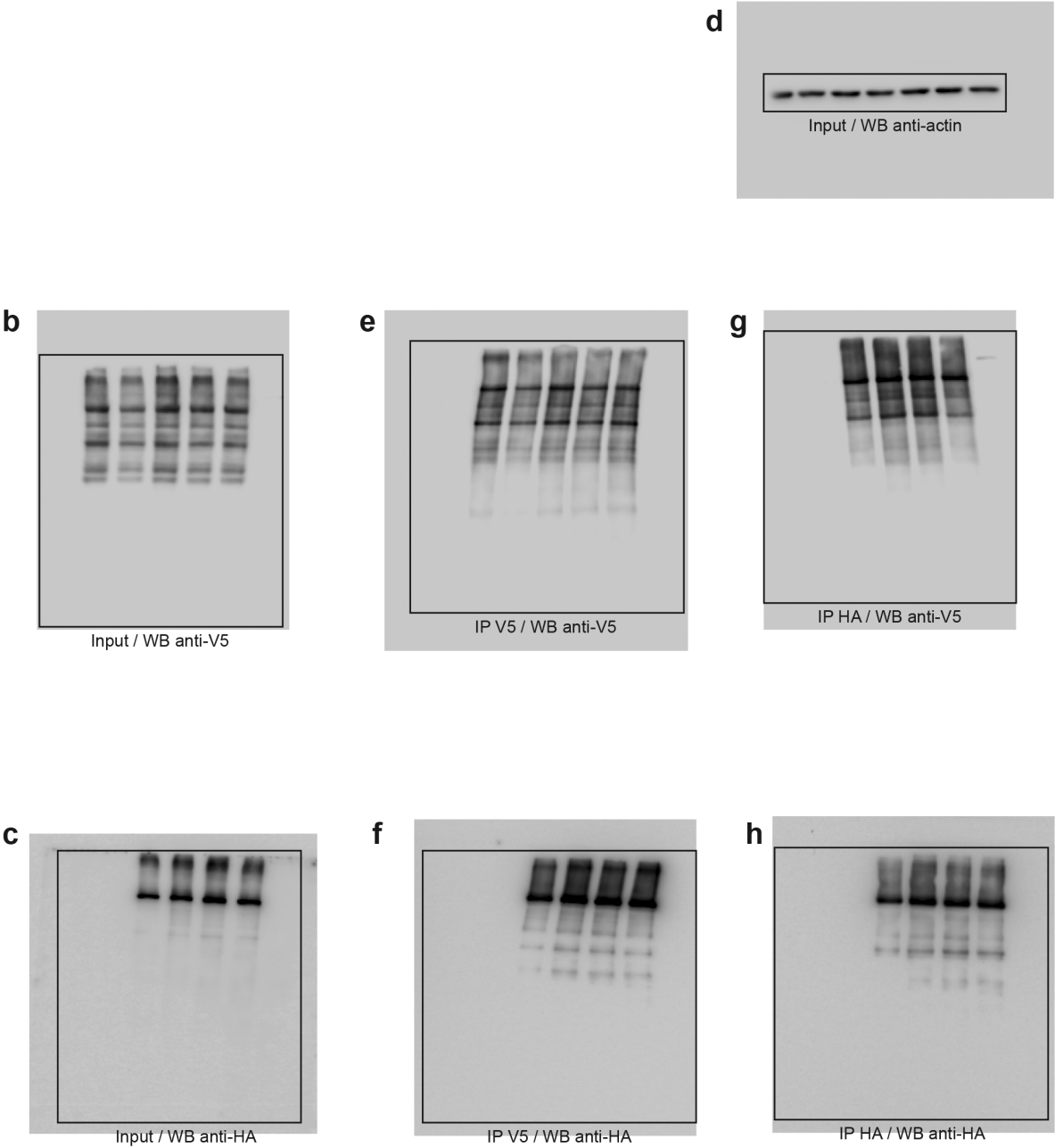

Raw images Figure 5

**b**

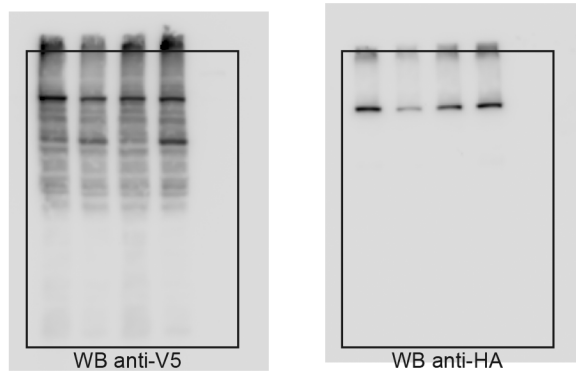

**c**

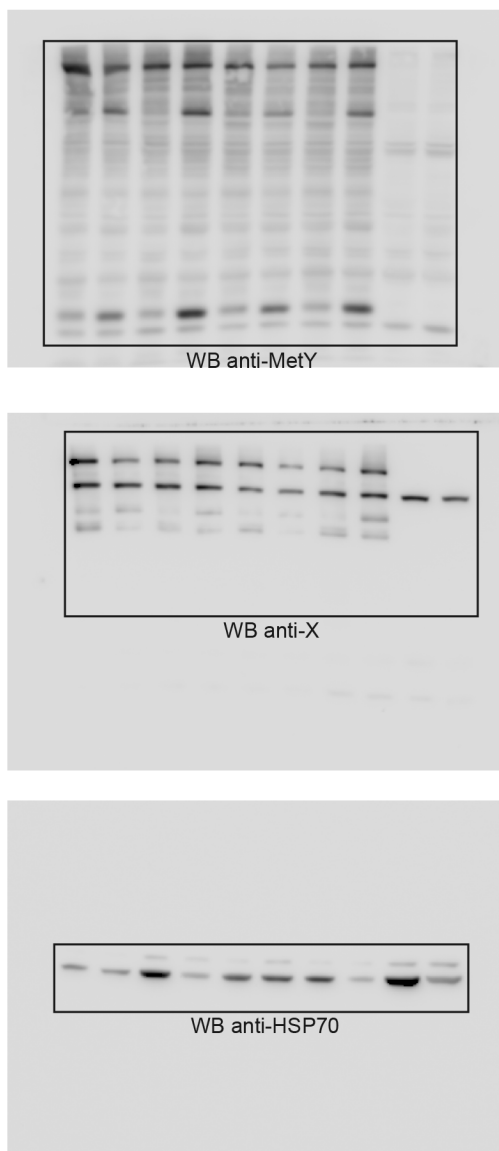

Supplement: S1 Data — File containing figures of the unprocessed gels used in figures 2–5. (PDF) [file pone.0343555.s003.pdf]
